# Data on isolation and purification of fibrinolytic enzyme from *Pseudomonas baetica* SUHU25

**DOI:** 10.1016/j.dib.2019.104369

**Published:** 2019-09-18

**Authors:** Asha S. Salunke, Arun S. Kharat

**Affiliations:** a Department of Biotechnology, Dr. Babasaheb Ambedkar Marathwada University, Subcampus Osmanabad, 413501, Maharashtra, India; b BioEra Life Sciences Pvt. Ltd., Survey Number 125, Mumbai – Bangalore Highway, Tathawade, Pune, 411033, Maharashtra, India; c Laboratory of Applied Microbiology, School of Life Sciences, Jawaharlal Nehru University, New Delhi, 110067, India

**Keywords:** *Pseudomonas baetica* SUHU25, Fibrinolytic, Blood clot, Enzyme, Cardiovascular disease

## Abstract

The present dataset provides methodology to isolate and purify fibrinolytic enzyme from microbe isolated from the natural source. The information provided in this data article includes (1) isolation and identification of *Pseudomonas baetica* SUHU25, (2) optimization of cultural conditions, (3) extraction and purification of fibrinolytic enzyme, (4) protein estimation, (5) assay of fibrinolytic activity, (6) SDS PAGE for purified enzyme protein, (7) effect of pH, temperature and metal ions on fibrinolytic activity of enzyme protein, and (8) In-vitro blood clot dissolution assay.

**Table:** Specifications Table

Subject	Microbiology
Specific subject area	Microbial enzymes, fibrinolytic enzymes from microbes
Type of data	Table Figure
How data were acquired	Isolation of *Pseudomonas baetica* SUHU25 was done from selective nutrient medium. Identification was achieved by DNA Extraction using Genomic DNA Extraction Kit (BioEra Life Sciences Pvt. Ltd., India), 16s rDNA gene amplification by PCR (BioEra Life Sciences Pvt. Ltd., India) and sequencing (ABI 3130 genetic analyzer and Big dye terminator version 3.1 cycle sequencing kit). Protein estimation and fibrinolytic enzyme protein assay was performed using spectrophotometer (Model ELITE, BioEra Life Sciences Pvt. Ltd., India). SDS PAGE to analyze molecular weight of purified enzyme proten performed using vertical gel electrophoresis system (BioEra Life Sciences Pvt. Ltd., India).
Data format	Raw
Parameters for data collection	Incubation temperature (°C), incubation time (hours), relative activities (%), concentrations (mM), molecular weight (kDa)
Description of data collection	The experimental data was obtained to optimize of cultural conditions, to extract and purify fibrinolytic enzyme, and to characterize fibrinolytic activity of purified enzyme protein from *Pseudomonas baetica* SUHU25. Also In-vitro application of the purified fibrinolytic enzyme was demonstrated by Blood clot dissolution assay
Data source location	BioEra Life Sciences Pvt. Ltd., Survey Number 125, Mumbai – Bangalore Highway, Tathawade, Pune - 411033, Maharashtra, India.
Data accessibility	Data is presented in this article

**Table:** 

**Value of the data** • There is extensive research in search of a effective fibrinolytic enzymes as presently available fibrinolytic enzymes are prone to low specificity. The presented data is first report of fibrinolytic enzyme from *Pseudomonas baetica* SUHU25. • The scientific fraternity looking for novel sources of fibrinolytic enzymes can use the presented data • A further study on characterization of fibrinolytic enzyme and testing efficacy of enzyme by other available In-vitro and In-vivo methods can be planned

## 1 Data

**Fig. 1:**
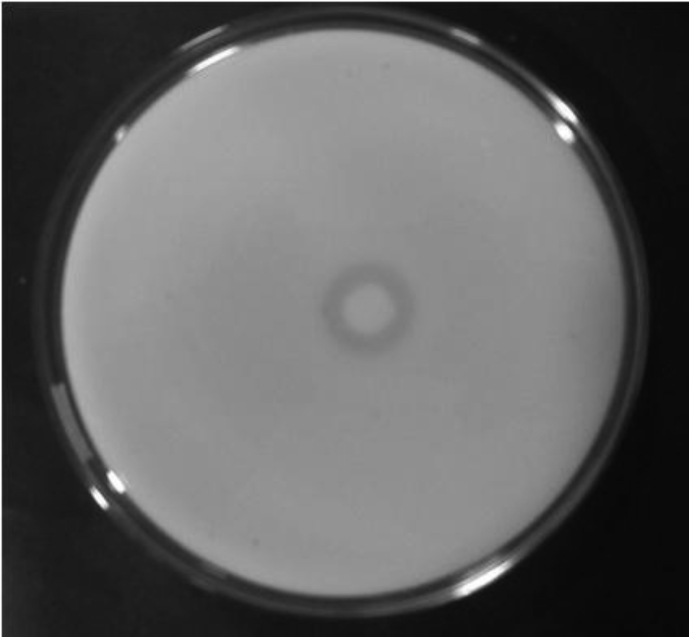
*Pseudomonas baetica* SUHU25 showing proteolytic activity on skim milk agar.

**Fig. 2:**
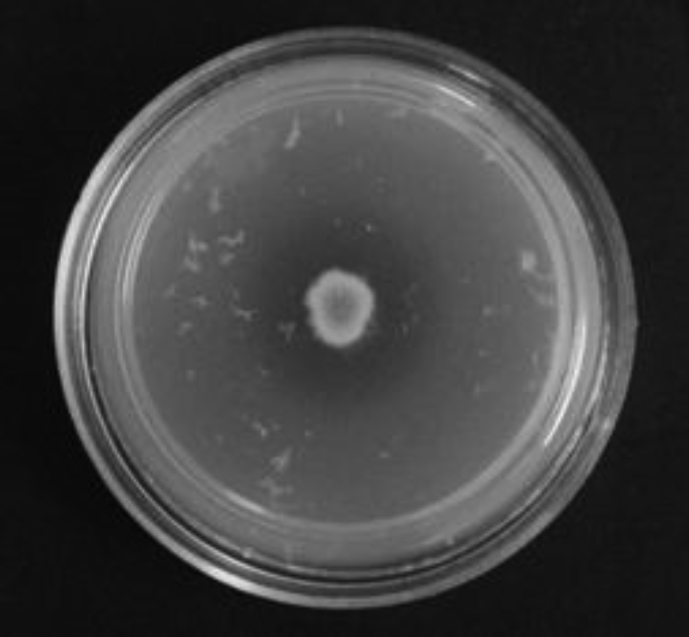
*Pseudomonas baetica* SUHU25 showing fibrinolytic activity on fibrin agar.

**Fig. 3:**
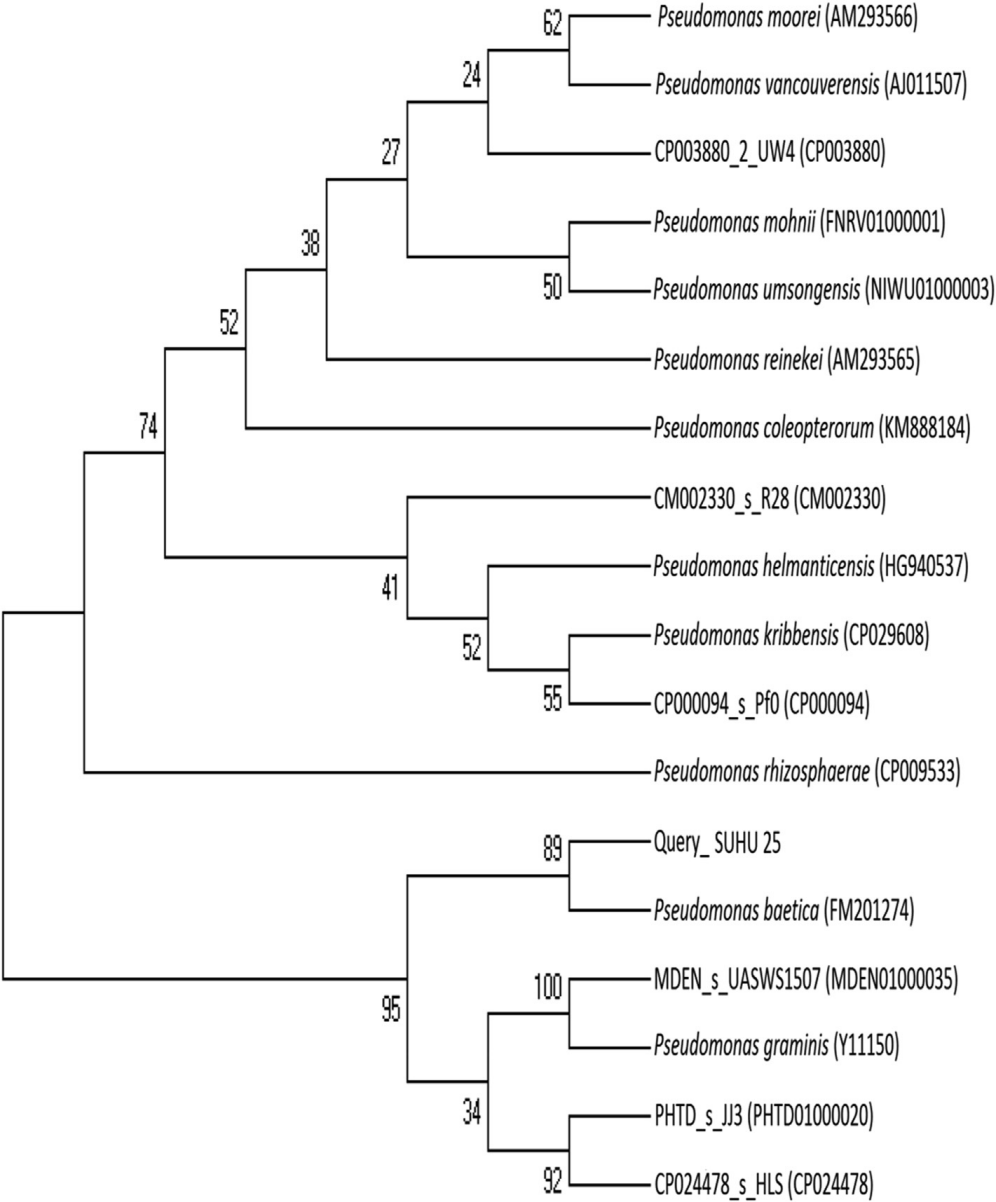
Phylogenetic tree of *Pseudomonas baetica* SUHU25 (NCBI Accesssion Number MK182973).

**Fig. 4:**
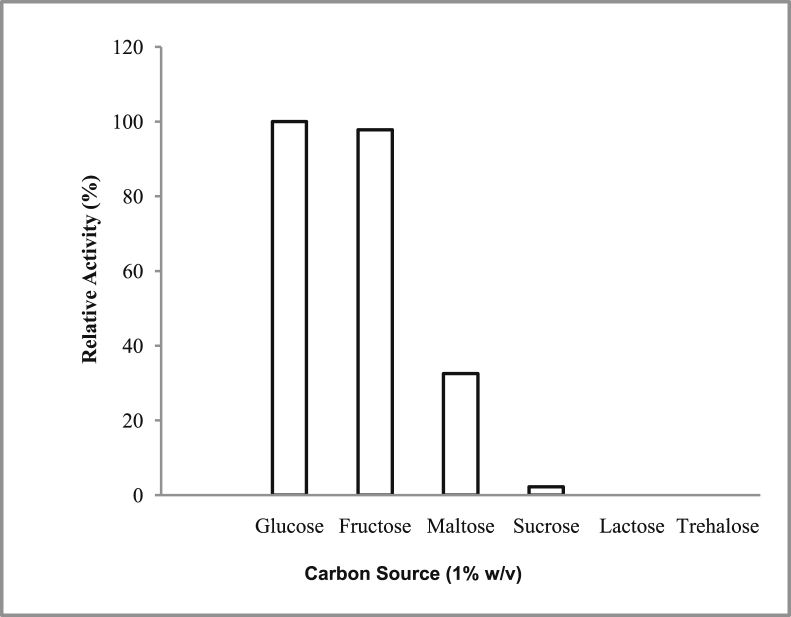
Effect of different carbon source on fibrinolytic activity of *Pseudomonas baetica* SUHU25.

**Fig. 5:**
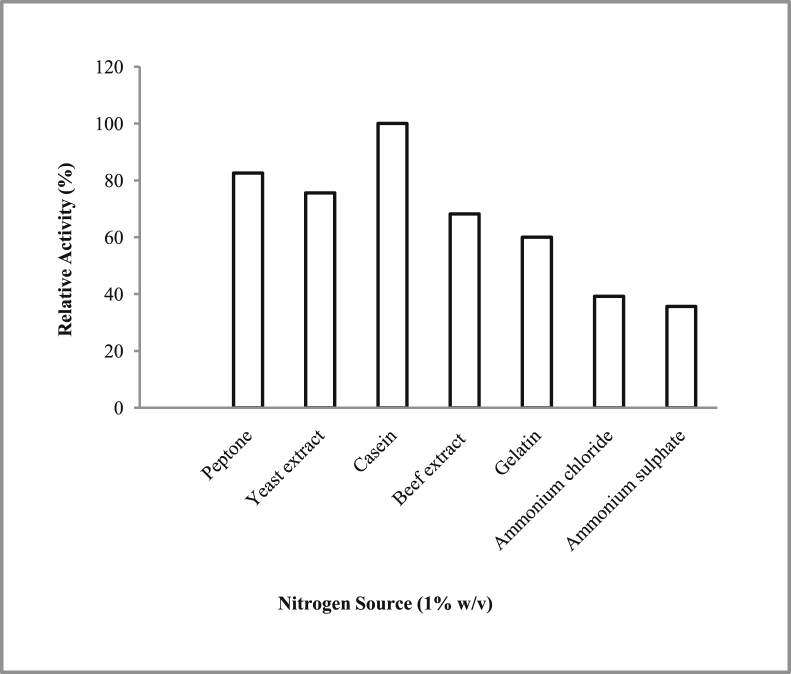
Effect of different nitrogen source on fibrinolytic activity of *Pseudomonas baetica* SUHU25.

**Fig. 6:**
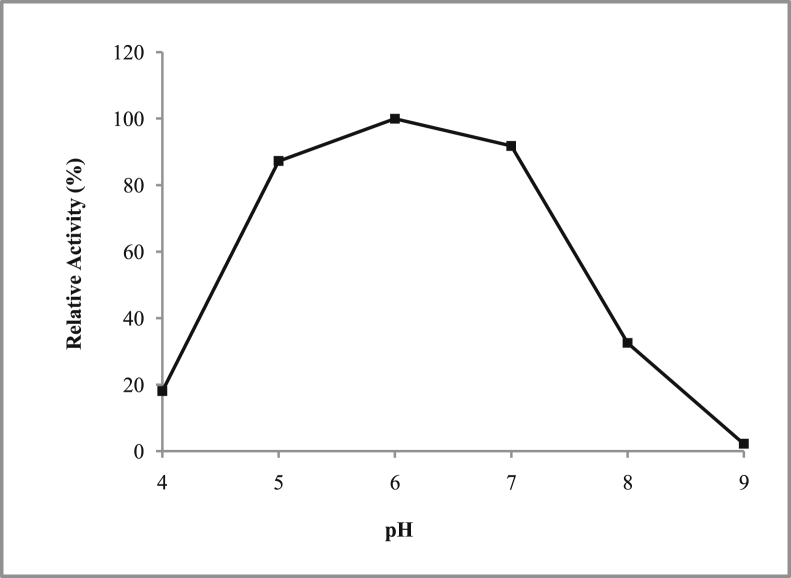
Effect of pH on fibrinolytic activity of *Pseudomonas baetica* SUHU25.

**Fig. 7:**
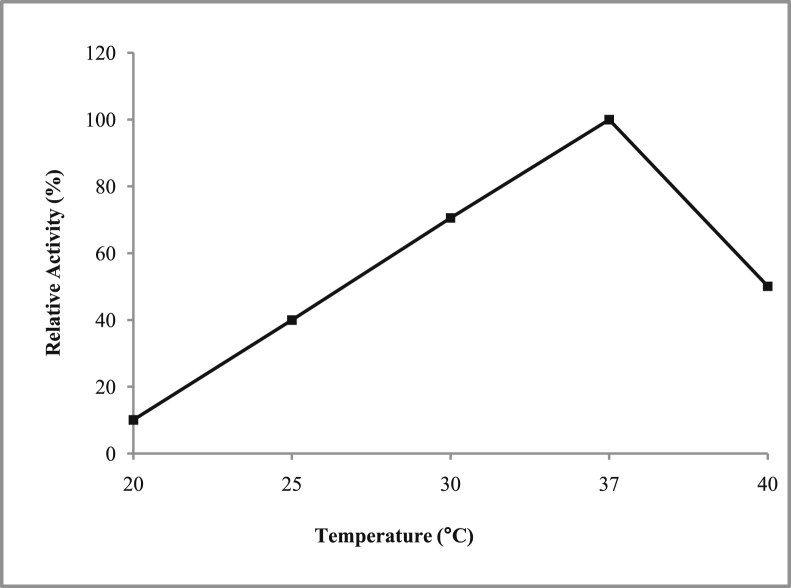
Effect of temperature on fibrinolytic activity of *Pseudomonas baetica* SUHU25.

**Fig. 8:**
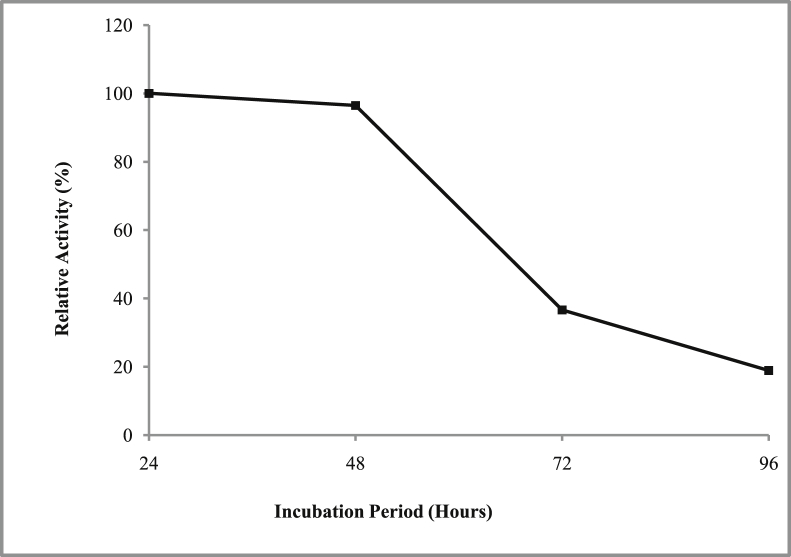
Effect of variable incubation period on fibrinolytic activity of *Pseudomonas baetica* SUHU25.

**Fig. 9:**
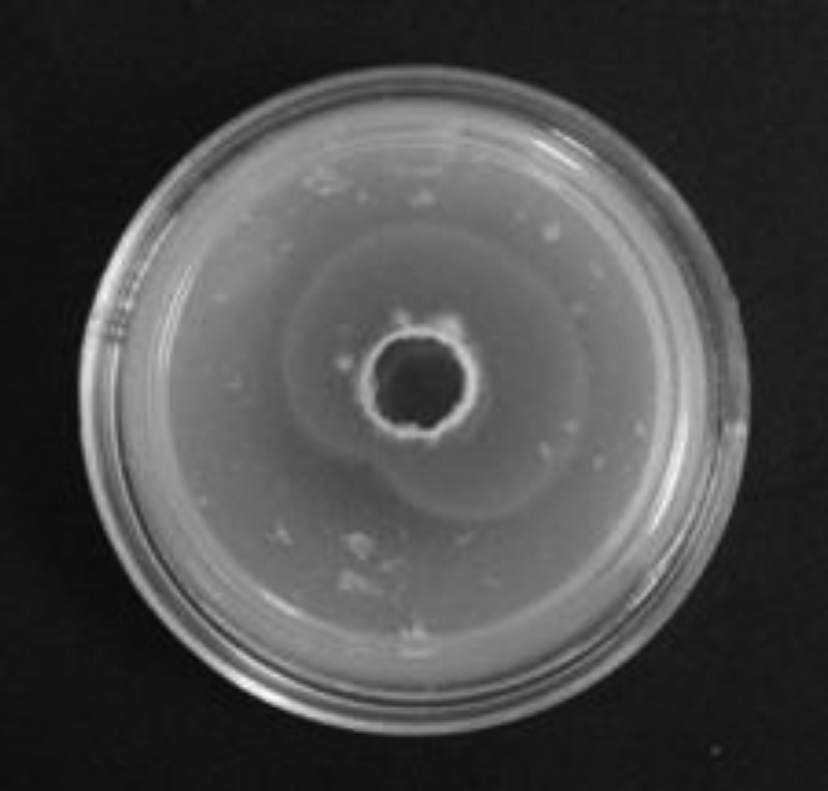
Fibrinolytic activity on fibrin agar plate by cell free media supernatant of *Pseudomonas baetica* SUHU25 grown in optimized cultural conditions.

**Fig. 10:**
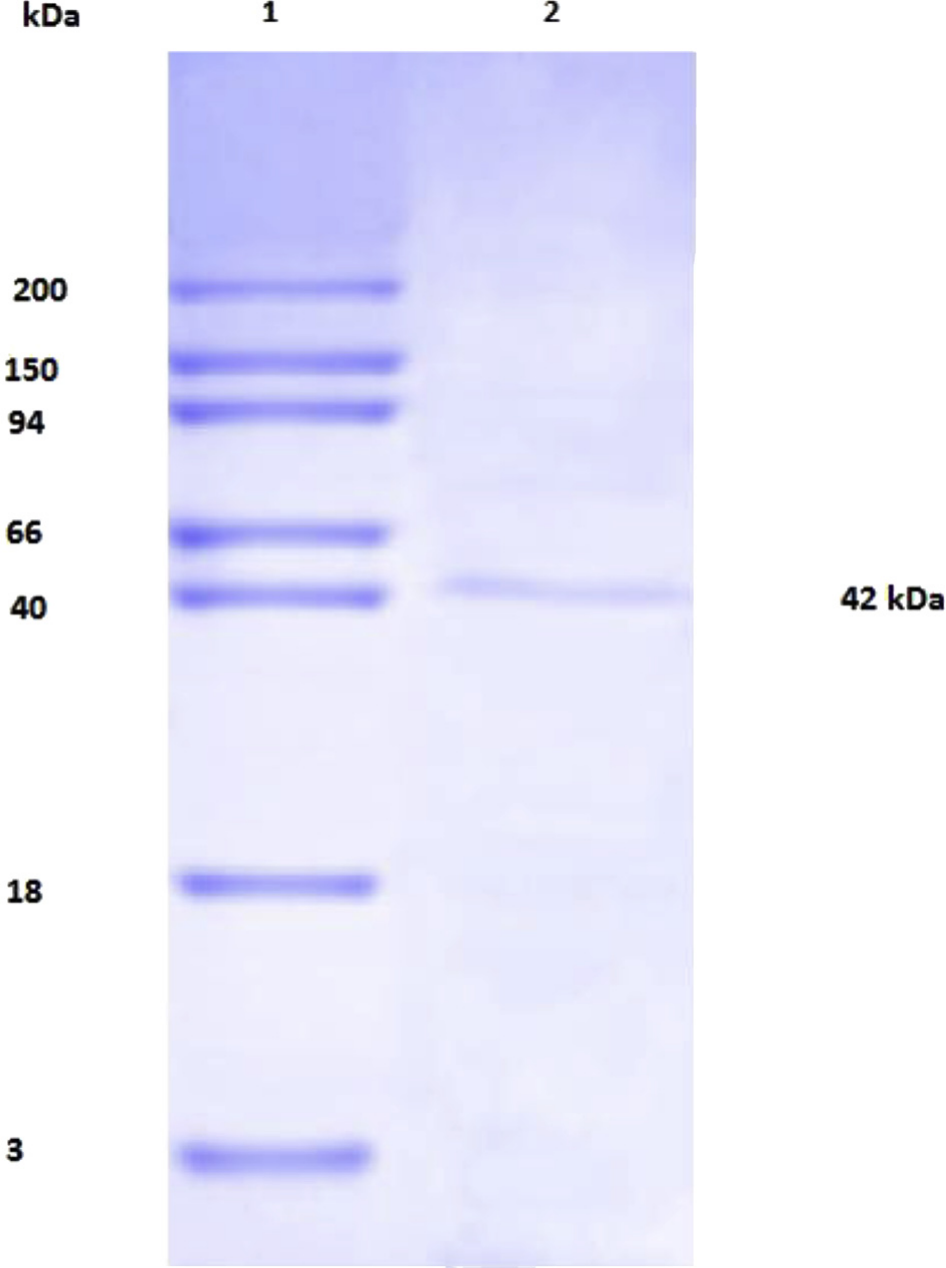
SDS PAGE of purified fibrinolytic enzyme from *Pseudomonas baetica* SUHU25. Lane 1 – Molecular weight marker; Lane 2 – Purified enzyme protein.

**Fig. 11:**
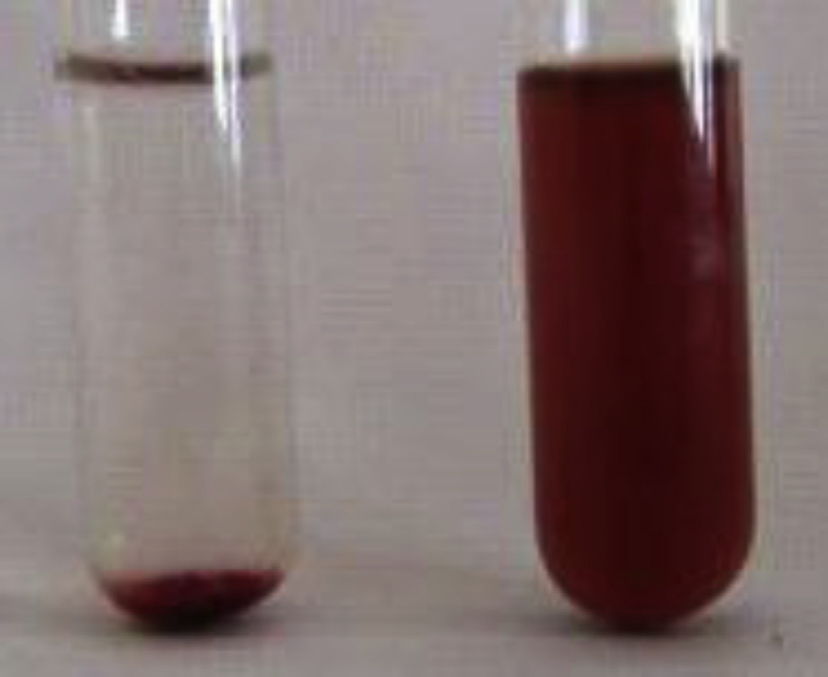
Lysis of Blood clot in-vitro by purified fibrinolytic enzyme preparation of *Pseudomonas baetica* SUHU25.

**Fig. 12:**
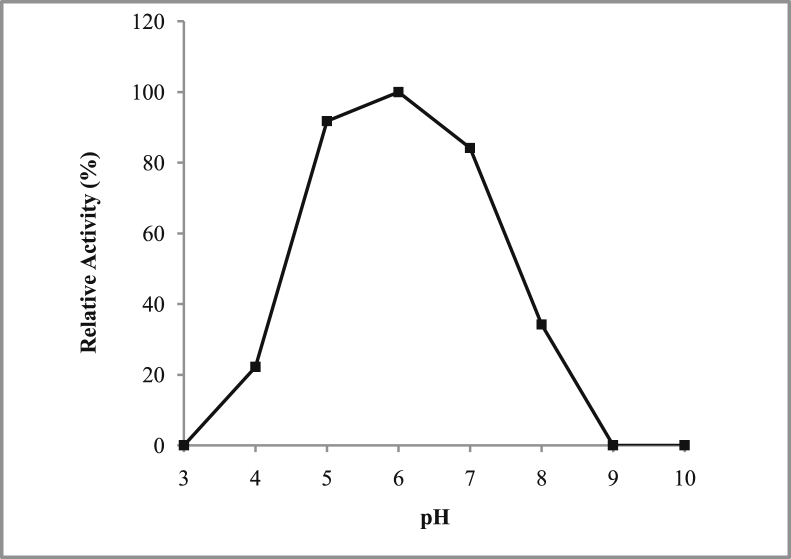
Effect of pH on purified fibrinolytic enzyme activity of *Pseudomonas baetica* SUHU25.

**Fig. 13:**
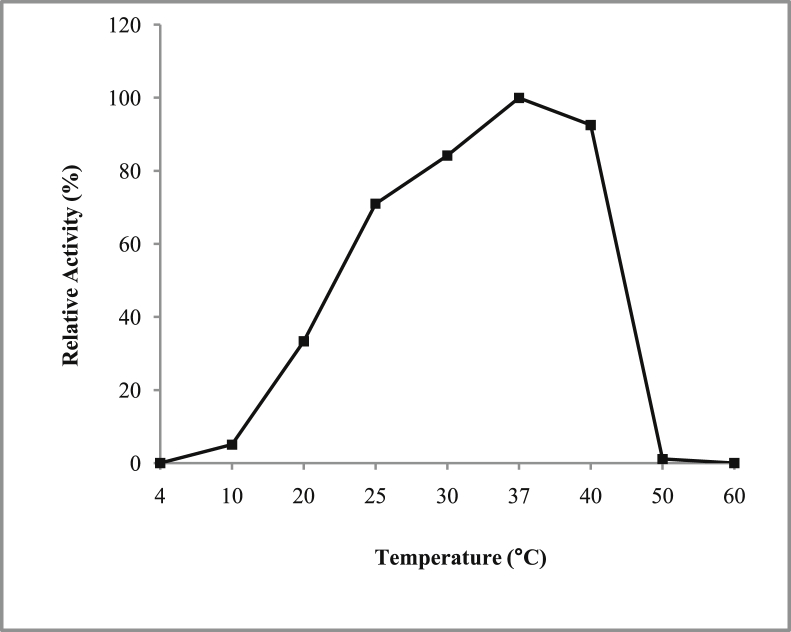
Effect of temperature on purified fibrinolytic enzyme activity of *Pseudomonas baetica* SUHU25.

**Fig. 14:**
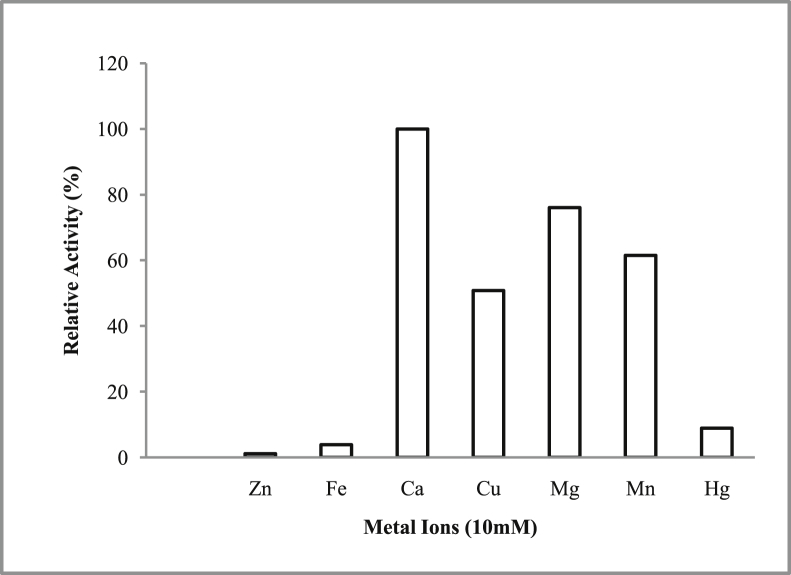
Effect of metal ions on purified fibrinolytic enzyme activity of *Pseudomonas baetica* SUHU25.

*Pseudomonas baetica* SUHU25 isolated from a local fish market showed proteolytic activity on skim milk agar as presented in Fig. 1 and fibrinolytic activity on fibrin agar in Fig. 2. Fig. 3 shows the Phylogenetic tree of *Pseudomonas baetica* SUHU25 obtained after 16s rRNA sequencing. Fig. 4, Fig. 5, Fig. 6, Fig. 7, Fig. 8 shows relative activity (%) of fibrinolytic enzyme of *Pseudomonas baetica* SUHU25 with change in carbon source, nitrogen source, pH, temperature and incubation period respectively. Fig. 9 shows fibrinolytic enzyme activity of cell free media supernatant of *Pseudomonas baetica* SUHU25. Fig. 10 represents the SDS PAGE of purified fibrinolytic enzyme from *Pseudomonas baetica* SUHU25. Fig. 11shows In-vitro blood clot dissolution by the purified enzyme preparation. Fig. 12, Fig. 13, Fig. 14 shows relative activity (%) of fibrinolytic enzyme with change in pH, temperature and presence of various metals respectively. Purification scheme of fibrinolytic enzyme form *Pseudomonas baetica* SUHU25 is detailed in Table 1.

**Table 1:** Purification of fibrinolytic enzyme from *Pseudomonas baetica* SUHU25.

Description	Total Activity (U)	Total Protein (mg)	Specific Activity (U/mg)	Yield (%)	Purification Fold
Cell free homogenate	4988.2	1566.4	3.18	100.0	1.00
Acetone Precipitation	2096.6	601.50	3.49	42.03	1.10
Anion exchange chromatography on DEAE Sephadex A 50	848.20	158.00	5.37	17.00	1.69
Gel filtration chromatography on Sephadex G 100	390.00	32.20	12.11	7.82	3.81

## 2 Experimental design, materials, and methods

### 2.1 Screening for fibrinolytic microorganisms

Sample was collected in sterile container from a local fish market at wash and waste disposal site in Pune, India for the isolation of potential fibrinolytic microbes. Primary Screening was done by serially diluting sample with physiological saline up to 10^−3^. 0.1 ml of dilution was plated on nutrient agar plates and incubated at 37 °C for 24 hours for microbial growth. Each well isolated bacterial colony after 24 hours of growth on nutrient agar was spot inoculated on skim milk agar for analyzing proteolytic activity. Cultures showing zone of clearance on skim milk agar were further spot inoculated on fibrin agar for 24 hours at 37 °C for secondary screening. Zone of clearance was noted after incubation for each culture [1].

### 2.2 Identification of fibrinolytic isolate

Isolated culture showing larger zone of clearance on fibrin agar plate was identified by 16s rRNA Sequencing. Genomic DNA was extracted from the isolates using BioEra's Genomic DNA extraction kit. 16s rDNA gene was amplified by forward primer 5′- AGAGTRTGATCMTYGCTWAC-3′ and reverse primer 5′CGYTAMCTTWTTACGRCT-3′ with the programme consisting of denaturation at 94 °C for 5 minutes and subsequent 35 cycles of denaturation at 94 °C for 30 seconds, annealing at 55 °C for 30 seconds, and extension at 72 °C for 2 minutes followed by final extension at 72 °C for 5 minutes. The sequence analysis was performed using the ABI 3130 genetic analyzer and Big Dye Terminator version 3.1 cycle sequencing kit. The amplified product sequence comparison with database was performed using BLAST through the NCBI server [2].

### 2.3 Optimization of cultural conditions

The isolated culture was grown in the mineral salt medium (g/L: KH_2_PO_4_, 0.42; K_2_HPO_4_, 0.375; (NH_4_)_2_SO_4_, 0.244; NaCl, 0.015; CaCl_2_.2H_2_O, 0.015; MgSO_4_.7H_2_O, 0.05; and FeCl_3_.6H_2_O, 0.054; pH 7 ± 0.1) supplemented with different carbon sources (such as 1% w/v glucose, fructose, maltose, sucrose, lactose and trehalose) and nitrogen sources (such as 1% w/v peptone, yeast extract, casein, beef extract, gelatin, ammonium chloride and ammonium sulphate). Effect of various physical parameters such as initial pH (4, 5, 6, 7, 8 and 9), temperature (20, 25, 30, 37, and 40 °C) and incubation period (24, 48, 72, and 96 hours) were checked. Optimization of variables was done by one-variable-at-a-time approach. All the experiments were conducted in triplicates.

### 2.4 Extraction and purification of fibrinolytic enzyme

The isolated culture was grown at 37 °C for 24 hours in the optimized nutrient medium (g/L: glucose, 10; casein, 10, KH_2_PO_4_, 0.42; K_2_HPO_4_, 0.375; NaCl, 0.015; CaCl_2_.2H_2_O, 0.015; MgSO_4_.7H_2_O, 0.05; and FeCl_3_.6H_2_O, 0.054; pH 6 ± 0.1). After incubation cells were separated from nutrient medium by centrifugation at 6000 rpm for 15 minutes at 4 °C. The fibrinolytic enzyme protein was extracted from the cell free supernatant by precipitation with five volumes of ice-cold acetone. Fibrinolytic enzyme from the acetone precipitated proteins was purified by anion exchange resin (DEAE Sephadex A50) and gel filtration chromatography on sephadex G100. All the steps were performed at 4 °C.

### 2.5 Protein estimation

Protein was estimated using dye-binding method described by Bradford [3].

### 2.6 Assay of fibrinolytic activity

Fibrinolytic activity was determined as reported by Tharwat et al. [4].

### 2.7 SDS PAGE for purified enzyme protein

SDS-PAGE was carried out to determine the purity and molecular weight of enzyme protein as described by Laemmli [5] using 10% polyacrylamide resolving gel. The gel was stained with Coomassie Brilliant Blue G 250 to visualize the protein bands.

### 2.8 Effect of pH and temperature on fibrinolytic activity of enzyme protein

The optimal pH for fibrinolytic activity of the enzyme was determined within pH range of 3–10 using acetate buffer (0.1 M, pH 3, 4 and 5), phosphate buffer (0.1 M, pH 6, 7 and 8), and Glycine buffer (0.1 M, pH 9 and 10). The effect of temperature was determined by measurement of residual activity of enzyme after incubation at different temperatures (4, 10, 20, 25, 30, 37, 40, 50 and 60 °C). 1 mg ml^−1^ of purified enzyme preparation was used for all reactions. The activity of enzyme was assayed as reported by Tharwat et al. [4].

### 2.9 Effect of metal ions on fibrinolytic activity of enzyme protein

The effect of presence of metal ions was determined by using MgCl_2_, ZnCl_2_, FeCl_3_, CuSO_4_, CaCl_2_, MnSO_4_, and HgCl_2_ at a final concentration of 10 mM. The purified preparation of enzyme at a concentration of 1 mg ml^−1^ was pre-incubated in both the presence and absence of each cation for 1 hour at 37 °C. The activity of enzyme was assayed as reported by Tharwat et al. [4].

### 2.10 In-vitro blood clot dissolution assay

In vitro blood clot analysis was carried out according to Avhad et al. [6] with some modifications. 5 ml of purified enzyme preparation added to 0.5 g human blood clot in a test tube and kept for incubation at 37 °C till the complete blood clot lyses.
