# Supplementary material for: Data on isolation and purification of fibrinolytic enzyme from Pseudomonas baetica SUHU25
Source: Data Brief. 2019 Sep 18;26:104369. doi: 10.1016/j.dib.2019.104369 (PMC6811981; doi:10.1016/j.dib.2019.104369)
Supplement: Multimedia component 1 [file mmc1.pdf]

_Pseudomonas baetica_strain SUHU25 16S ribosomal RNA gene, partial sequence

FASTA Sequence

Figure S1. Accession Number MK182973

>MK182973.1 *Pseudomonas baetica* strain SUHU25 16S ribosomal RNA gene, partial sequence

GCCTGGTAGTGGGGGACAACGTTTCGAAAGGAACGCTAATACCGCATACGTCCTACGGGAGAAAGCAGGG

GACCTTCGGGCCTTGCGCTATCAGATGAGCCTAGGTCGGATTAGCTAGTTGGTGAGGTAATGGCTCACCA

AGGCGACGATCCGTAACTGGTCTGAGAGGATGATCAGTCACACTGGAACTGAGACACGGTCCAGACTCCT

ACGGGAGGCAGCAGTGGGGAATATTGGACAATGGGCGAAAGCCTGATCCAGCCATGCCGCGTGTGTGAAG

AAGGTCTTCGGATTGTAAAGCACTTTAAGTTGGGAGGAAGGGCAGTAAGCGAATACTTTGCTGTTTTGAC

GTTACCGACAGAATAAGCACCGGCTAACTCTGTGCCAGCAGCCGCGGTAATACAGAGGGTGCAAGCGTTA

ATCGGAATTACTGGGCGTAAAGCGCGCGTAGGTGGTTTGTTAAGTTGGATGTGAAATCCCCGGGCTCAAC

CTGGGAACTGCATTCAAAACTGGCGAGCTAGAGTAGGGCAGAGGGTGGTGGAATTTCCTGTGTAGCGGTG

AAATGCGTAGATATAGGAAGGAACACCAGTGGCGAAGGCGACCACCTGGACTGATACTGACACTGAGGTG

CGAAAAGGTGGGGAGCAAACAGGATTAGATACCCTGGTAGTCCACGCCGTAAACGATGTCAACTAGCCGT

TGGGAGCCTTGAGCTCTTAGTGGCGCAGCTAACGCATTAAGTTGACCGCCTGGGGAGTACGGCCGCAAGG

TTAAAACTCAAATGAATTGACGGGGGCCCGCACAAGCGGTGGAGCATGTGGTTTAATTCGAAGCAACGCG

AAGAACCTTACCAGGCCTTGACATCCAATGAACTTTCCAGAGATGGATTGGTGCCTTCGGGAACATTGAG

ACAGGTGCTGCATGGCTGTCGTCAGCTCGTGTCGTGAGATGTTGGGTTAAGTCCCGTAACGAGCGCAACC

CTTGTCCTTAGTTACCAGCACGTTATGGTGGGCACTCTAAGGAGACTGCCGGTGACAAACCGGAGGAAGG

TGGGGATGACGTCAAGTCATCATGGCCCTTACGGCCTGGGCTACACACGTGCTACAATGGTCGGTACAAA

GGGTTGCCAAGCCGCGAGGTGGAGCTAATCCCATAAAACCGATCGTAGTCCGGATCGCAGTCTGCAACTC

GACTGCGTGAAGTCGGAATCGCTAGTAATCGCGAATCAGAATGTCGCGGTGAATACGTTCCCGGGCCTTG

TACACACCGCCCGTCACACCATGGGAGTGGGTTGCACCAGAAG

Table S1. Data on Effect of different carbon source on fibrinolytic activity of *Pseudomonas baetica* SUHU25 (With reference to figure 4)

**Carbon Source (1% w/v)**	**Relative Activity (%)**
Glucose	100
Fructose	97.78
Maltose	32.5
Sucrose	2.2
Lactose	0
Trehalose	0

Table S2. Data on Effect of different nitrogen source on fibrinolytic activity of *Pseudomonas baetica* SUHU25 (With reference to figure 5)

**Nitrogen Source (1% w/v)**	**Relative Activity (%)**
Peptone	82.56
Yeast extract	75.6
Casein	100
Beef extract	68.2
Gelatin	60
Ammonium chloride	39.2
Ammonium sulphate	35.66

Table S3. Data on Effect of pH on fibrinolytic activity of *Pseudomonas baetica* SUHU25 (With reference to figure 6)

pH	Relative Activity (%)
4	18.07
5	87.26
6	100
7	91.84
8	32.58
9	2.22

Table S4. Data on Effect of temperature on fibrinolytic activity of *Pseudomonas baetica* SUHU25 (With reference to figure 7)

Temperature (°C)	Relative Activity (%)
20	10.1
25	40
30	70.56
37	100
40	50.12

Table S5. Data on Effect of incubation period on fibrinolytic activity of *Pseudomonas baetica* SUHU25 (With reference to figure 8)

**Incubation Time (hours)**	**Relative Activity (%)**
24	100
48	96.44
72	36.62
96	18.86

Table S6. Data on Effect of pH on purified fibrinolytic enzyme activity of *Pseudomonas baetica* SUHU25 (With reference to figure 12)

**pH**	**Relative Activity (%)**
3	0
4	22.2
5	91.8
6	100
7	84.2
8	34.2
9	0
10	0

Table S7. Data on Effect of temperature on purified fibrinolytic enzyme activity of *Pseudomonas baetica* SUHU25 (With reference to figure 13)

**Temperature (°C)**	**Relative Activity (%)**
4	0
10	5.06
20	33.34
25	71.02
30	84.22
37	100
40	92.56
50	1.12
60	0

Table S8. Data on Effect of metal ions on purified fibrinolytic enzyme activity of *Pseudomonas baetica* SUHU25 (With reference to figure 14)

**Metal Ion (10mM)**	**Relative Activity (%)**
Zn	1.12
Fe	3.88
Ca	100
Cu	50.82
Mg	76.06
Mn	61.52
Hg	8.9
